# The Role of TRPV1/CGRP Pathway Activated by *Prevotella melaninogenica* in Pathogenesis of Oral Lichen Planus

**DOI:** 10.3390/ijms26020662

**Published:** 2025-01-14

**Authors:** Pan Xu, Ruru Shao, Pingyi Zhu, Jian Fei, Yuan He

**Affiliations:** 1 Shanghai Engineering Research Center of Tooth Restoration and Regeneration & Tongji Research Institute of Stomatology & Department of Oral Mucosal Diseases, Shanghai Tongji Stomatological Hospital and Dental School, Tongji University, Shanghai 200072, China; xupan202009@tongji.edu.cn (P.X.); shaorurusrr@163.com (R.S.); 2131264@tongji.edu.cn (P.Z.); 2 School of Life Science and Technology, Tongji University, Shanghai 200072, China

**Keywords:** oral lichen planus, TRPV1, *Prevotella melaninogenica*, CGRP, IL-36γ

## Abstract

The distinctive clinicopathologic characteristics of OLP indicated that both microbial dysbiosis and neurogenic inflammation may be jointly involved in its progression, and transient receptor potential vanilloid receptor-1 (TRPV1) may be a crucial element. The purpose of this study was to explore how TRPV1 mediated *P. melaninogenica*-induced inflammation. Meanwhile, we aimed to unravel how IL-36γ dysregulated the barrier function in oral keratinocytes. Here, the expression of TRPV1, calcitonin gene-related peptide (CGRP), and its receptor receptor activity-modifying protein 1 (RAMP1) in OLP patients were detected. *Prevotella melaninogenica* (*P. melaninogenica*) was used to build a mouse model of oral chronic inflammation. Normal human oral keratinocytes (NHOKs) stimulated by *P. melaninogenica* were used to examine TRPV1 activation and CGRP release. To investigate the effect of exogenous CGRP on Interleukin-36 gamma (IL-36γ) expression in NHOKs and bacterial viability, *P. melaninogenica* and NHOKs were treated with it, respectively. Recombinant IL-36γ protein was used to probe its regulation of oral epithelial barrier function. TRPV1, CGRP, and RAMP1 were substantially expressed in OLP. *P. melaninogenica* increased TRPV1 expression in mice and caused the release of CGRP and an increase in pro-inflammatory cytokines via activating TRPV1 in NHOKs. Blockade of TRPV1 suppressed *P. melaninogenica*-induced inflammation. CGRP boosted the production of IL-36γ released by NHOKs, resulting in lower expression of zonula occludens-1 (ZO-1). Also, CGRP can decrease the viability of *P. melaninogenica*. Together, these findings provide fresh insight into the vital role performed by *P. melaninogenica*-induced functional changes in oral epithelial cells and neurons in an intricate OLP inflammatory process.

## 1. Introduction

Oral lichen planus (OLP) is an immune-mediated chronic inflammatory disease of the oral mucosa (commonly in buccal mucosa) with unknown etiology. The pathological characteristic features of OLP include the apoptosis of basal keratinocytes and the activation and significant accumulation of T-lymphocytes within the lamina propria [1]. Unraveling its pathogenesis is important for the treatment of OLP.

Microorganisms have been identified as probable contributors to the development of OLP [2]. Several studies have found microbial dysbiosis in both saliva and tissue samples from OLP patients [3,4,5,6,7]. Closely related to the pathogenesis of OLP, oral bacteria have been implicated in the disruption of the oral epithelial barrier, invasion of the lamina propria, internalization of oral epithelial cells and T-cells, and stimulation of chemokine production [4]. One of the first microorganisms to colonize the oral cavity is *Prevotella melaninogenica* (*P. melaninogenica*), a black-pigmented anaerobic bacterium from the genus *Prevotella* in the phylum *Bacteroidota* [8]. *P. melaninogenica* has been found to be more abundant in a number of oral disorders, including dental caries [9], periodontitis [10], peri-implantitis [11], Sjӧgren syndrome [12], and oral squamous cell carcinoma (OSCC) [13]. In our preliminary study, *P. melaninogenica* was found to be abundant in the buccal mucosa of OLP patients, and further research revealed that it could invade the oral epithelium and even the lamina propria in OLP [14]. Moreover, *P. melaninogenica* promoted the expression of IL-36γ (a pro-inflammatory cytokine in OLP) in oral keratinocytes [15]. All things considered, it appears possible that *P. melaninogenica* may be a contributing factor to the chronic inflammatory process of OLP.

Neurogenic inflammation refers to inflammatory responses triggered by the activation of primary sensory neurons and the subsequent release of inflammatory neuropeptides, including substance P (SP) and calcitonin gene-related peptide (CGRP) [16]. The oral mucosa is highly innervated by sensory nerves (fiber types Aδ and C) from trigeminal divisions [17,18]. Many OLP-related distinct clinicopathological characteristics indicate that neurogenic inflammation may play an important role in OLP progression. For example, most OLP lesions tend to appear in both cheeks in a symmetrical style and are distributed along the buccinator nerve (third trigeminal division), suggesting that the pathogenesis of OLP may be related to the cross-reflection of the nerves [18]. OLP lesions may be characterized by local pain or paresthesia, such as woody astringency, burning, itchiness, etc., which are exacerbated by spicy, hot, sour, salty, or other irritating foods [19,20], suggesting that sensory nerves have been activated. According to research, there were also more nerve fibers distributed, and the distribution was denser in OLP lesions than in normal mucosa [21]. However, less research has been performed on the connection between neurogenic inflammation and the development of OLP.

Transient receptor potential vanilloid receptor-1 (TRPV1), a non-selective cation channel, was triggered by several stimuli, such as noxious heat (>~43 °C) [22], acidic pH [23], protons [24], and toxins [25]. TRPV1 could induce neurogenic inflammation by promoting the release of neuropeptides SP and CGRP upon activation [26]. Upon activation in neurons, TRPV1 promoted the release of neuropeptides CGRP and SP. By interacting with their receptors on a range of cells, such as mast cells, macrophages, and T lymphocytes, the neuropeptides produced pro-inflammatory effects and triggered an immunological response [27,28,29]. The oral mucosa may release CGRP and SP locally when exogenous stimuli activate TRPV1, transient receptor potential ankyrin 1 (TRPA1), and transient receptor potential ankyrin melastatin 8 (TRPM8) on trigeminal sensory nerves, which can ultimately lead to neurogenic inflammation [30]. TRPV1 also exerted an important role in non-neuronal cells. In skin keratinocytes, ultraviolet B irradiation promoted the expression of keratin 1 and keratin 10 by activating TRPV1 [31]. TRPV1 was decreased in human epidermal keratinocytes exposed to low temperature (25 °C), and the induction of IL-1β and thymic stromal lymphopoietin by low temperature was blocked due to the loss of TRPV1 [32]. In our preliminary research, *P. melaninogenica* promoted the expression of TRPV1 when it invaded oral keratinocytes. This finding implied a potential connection between *P. melaninogenica* and the TRPV1 channel in oral keratinocytes [15].

There were also studies showing that enhanced IL-36 signaling exacerbated defective mucosal repair in the intestine [33]. IL-36γ increased the epithelial and endothelial permeability in cultured sinonasal epithelial and endothelial cells [34]. Since barrier dysfunction may be an important part of OLP pathogenesis [35,36], the role of IL-36γ in the oral epithelial barrier needs to be explored further.

In this study, we hypothesized that TRPV1 may act as a sensor of pain and exogenous stimuli in oral mucosa and its over-expression could elicit chronic inflammation directly and indirectly. The purpose of this study was to explore how TRPV1 mediates *P. melaninogenica*-induced inflammation. Meanwhile, we aimed to unravel how IL-36γ dysregulates the barrier function in oral keratinocytes. This study sheds new light on the role of *P. melaninogenica*-mediated crosstalk between oral keratinocytes and neurons in OLP pathogenesis.

## 2. Results

### 2.1. TRPV1 Was Elevated in OLP and Facilitated Oral Mucosal Inflammation

#### 2.1.1. TRPV1 Was Elevated in Epithelium of OLP

**Figure 1:**
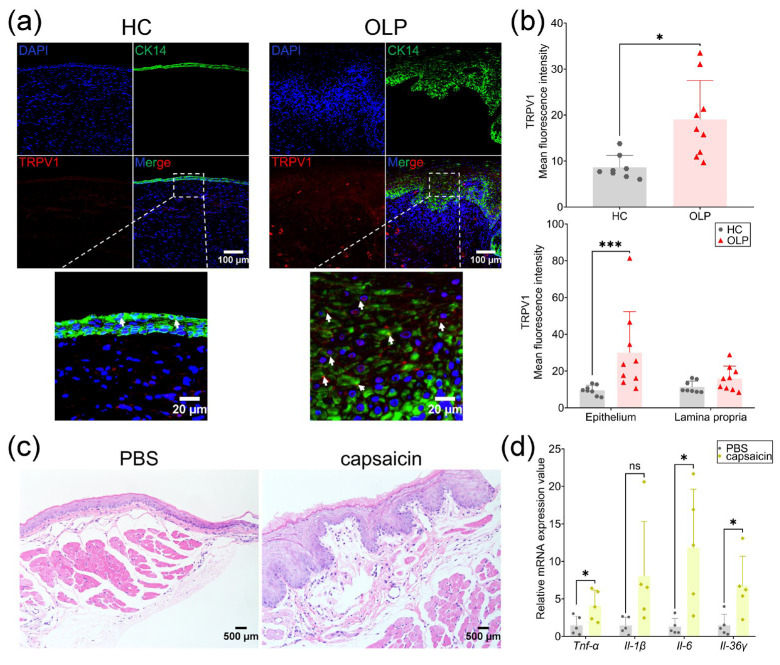
TRPV1 activation by capsaicin-induced oral mucosal inflammation. (**a**) Representative immunofluorescence images of oral mucosal sections using CK14 and TRPV1 antibodies in healthy controls (HC) (*n* = 8) and oral lichen planus (OLP) lesions (*n* = 9) and the quantitative analysis of TRPV1 (**b**). White arrows: TRPV1^+^CK14^+^ oral keratinocytes; (**c**) HE images of buccal mucosal sections from PBS- or capsaicin-treated mice; (**d**) the expression of pro-inflammatory cytokines in buccal mucosa of PBS- (*n* = 5) or capsaicin-treated mice (*n* = 5). * *p* < 0.05, *** *p* < 0.001, ns: not significant.

To investigate TRPV1 expression in OLP, human samples were collected, and immunofluorescence was performed. TRPV1 was highly expressed in OLP compared to healthy controls (HCs) (Figure 1a). Of note, TRPV1 was co-located with cytokeratin 14 (CK14), the marker of the basal oral keratinocytes, and its higher fluorescence intensity was seen in the epithelium of OLP samples but not in the lamina (Figure 1a,b).

#### 2.1.2. TRPV1 Activation Promoted Oral Mucosal Inflammation

Next, we injected the TRPV1 agonist capsaicin into the mouse buccal mucosa. Increased inflammatory infiltration was seen following capsaicin stimulation (Figure 1c). Also, capsaicin evoked a significant elevation of the mRNA levels of pro-inflammatory cytokines, including *Tnf-α*, *Il-6,* and *Il-36γ* (Figure 1d). Although the increased expression of *Il-1β* was seen, it did not reach a statistically significant difference (Figure 1d). From these, it was indicated that TRPV1 activation by capsaicin can promote oral mucosal inflammation.

### 2.2. P. melaninogenica Invasion of the Oral Mucosa Promoted TRPV1 Expression Both in the Local Mucosa and in the Trigeminal Ganglion

#### 2.2.1. *P. melaninogenica* Promoted TRPV1 Expression in the Local Oral Mucosa

**Figure 2:**
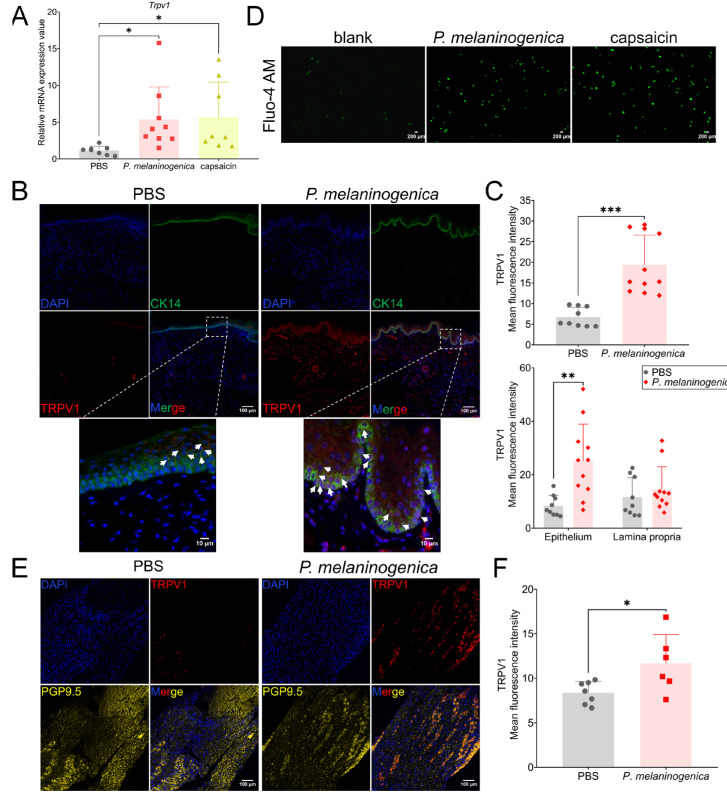
*P. melaninogenica* promoted TRPV1 activation in oral mucosa. (**A**) The mRNA expression of *Trpv1* in buccal mucosa of mice inoculated with PBS (*n* = 7) or *P. melaninogenica* (*n* = 9). Capsaicin inoculation was used as a positive control (*n* = 8); (**B**) representative immunofluorescence images of buccal mucosal sections using CK14 and TRPV1 antibodies from mice inoculated by PBS (*n* = 9) or *P. melaninogenica* (*n* = 11) and the quantitative analysis of TRPV1 (**C**). White arrows: TRPV1^+^CK14^+^ oral keratinocytes; (**D**) intracellular Ca^2+^ concentration was detected using Fluo-4 AM in NHOKs stimulated by *P. melaninogenica*. Capsaicin group was used as a positive control; (**E**) representative immunofluorescence images of trigeminal ganglia sections using PGP9.5 and TRPV1 antibodies from mice inoculated with PBS (*n* = 7) or *P. melaninogenica* (*n* = 6) and the quantitative analysis of TRPV1 (**F**). * *p* < 0.05, ** *p* < 0.01, *** *p* < 0.001, ns: not significant.

We previously reported that *P. melaninogenica* was abundant in OLP [14], and, in this study, we further found the potential association between *P. melaninogenica* and TRPV1 by inoculating *P. melaninogenica* into the buccal mucosa of mice. The mRNA level of *Trpv1* was markedly elevated after inoculation with *P. melaninogenica* (Figure 2A). Similar to its expression in OLP (Figure 1a), TRPV1 was highly expressed in the buccal mucosa of *P. melaninogenica*-inoculated mice compared to the PBS group, and its co-localization with CK14 was more frequent in *P. melaninogenica* group (Figure 2B,C). We then wondered whether *P. melaninogenica* could activate TRPV1 in NHOKs; Ca^2+^ imaging showed that *P. melaninogenica* increased intracellular calcium concentration after *P. melaninogenica* co-culturing with NHOKs for 4 h at MOI 5 (Figure 2D).

#### 2.2.2. *P. melaninogenica* Promoted TRPV1 Expression in the Trigeminal Ganglion

To deeply investigate whether *P. melaninogenica* abundance in the oral mucosa could affect TRPV1 expression in the trigeminal ganglion via local nerve endings, we isolated the trigeminal ganglia of mice and performed immunofluorescence using TRPV1 and PGP9.5 (a neuronal and nerve fiber marker) antibodies. As shown in Figure 2E,F, the mean fluorescence intensity of TRPV1 in the trigeminal ganglia of mice in the *P. melaninogenica* group was increased, and its co-localization with PGP9.5 was also increased, compared to the PBS group. This also suggested that *P. melaninogenica* may stimulate local nerve endings in the oral mucosa and activate TRPV1 in the trigeminal ganglia.

### 2.3. Blocking TRPV1 Significantly Reduced P. melaninogenica-Induced Local Inflammation in the Oral Mucosa

**Figure 3:**
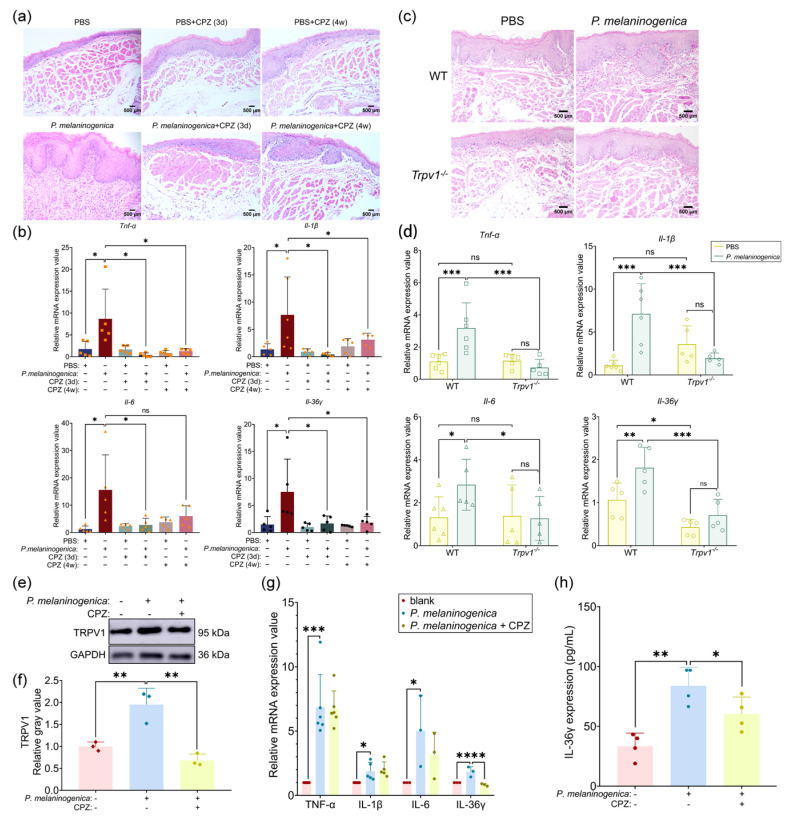
Blockade of TRPV1 attenuated *P. melaninogenica*-induced inflammation. (**a**) HE images of buccal mucosal sections from *P. melaninogenica*- and/or CPZ-treated mice; (**b**) the mRNA expression of pro-inflammatory cytokines in buccal mucosa of *P. melaninogenica*- and/or CPZ-treated mice by qPCR. The number of symbols in the column represents the number of samples (*n* = 5/6); (**c**) HE images of buccal mucosal sections from PBS- or *P. melaninogenica*-treated wild type (WT) mice and *Trpv1^−/−^* mice; (**d**) the mRNA expression of pro-inflammatory cytokines in buccal mucosa of PBS- or *P. melaninogenica*-treated WT mice and *Trpv1^−/−^* mice. The number of symbols in the column represents the number of samples (*n* = 5/6); (**e**) verification of the protein expression of TRPV1 in NHOKs stimulated by CPZ and/or *P. melaninogenica* and the quantitative analysis of TRPV1 (**f**); (**g**) the mRNA expression of pro-inflammatory cytokines in NHOKs stimulated by CPZ and/or *P. melaninogenica* by qPCR; (**h**) the protein expression of IL-36γ in NHOKs stimulated by CPZ and/or *P. melaninogenica* by ELISA. * *p* < 0.05, ** *p* < 0.01, *** *p* < 0.001, ns: not significant.

To investigate whether *P. melaninogenica* could trigger an inflammatory response in the oral mucosa and the role of TRPV1 in the process, CPZ was used to block TRPV1. As shown in Figure 3a, *P. melaninogenica* significantly enhanced inflammatory infiltration in the buccal mucosa of mice, which was greatly decreased by the simultaneous administration of CPZ. Meanwhile, *P. melaninogenica* boosted higher mRNA expression of pro-inflammatory cytokines in oral mucosa, which was greatly reversed by the administration of CPZ (Figure 3b). Also, CPZ itself did not cause inflammatory infiltration or the expression elevation of pro-inflammatory cytokines (Figure 3a,b). Then, we injected *P. melaninogenica* into *Trpv1^−/−^* mice and littermate WT mice. Compared to the *P. melaninogenica*-inoculated WT group, *Trpv1^−/−^* mice showed significant attenuation of the inflammatory infiltration after *P. melaninogenica* inoculation (Figure 3c). Meanwhile, the increased mRNA level of pro-inflammatory cytokines induced by *P. melaninogenica* was greatly alleviated after *Trpv1* knockdown (Figure 3d). Further, we validated this in NHOKs. CPZ significantly reduced *P. melaninogenica*-induced TRPV1 protein expression (Figure 3e,f). The mRNA and protein levels of inflammatory cytokines were tested, and it was found that *P. melaninogenica* promoted the expression of TNF-α, IL-1β, IL-6, and IL-36γ and that the IL-36γ concentration was significantly decreased following CPZ administration (Figure 3g,h). Together, these findings showed that TRPV1 can mediate *P. melaninogenica*-induced oral mucosal inflammatory responses, most likely by regulating IL-36γ.

### 2.4. P. melaninogenica Promoted CGRP Release and RAMP1 Expression

**Figure 4:**
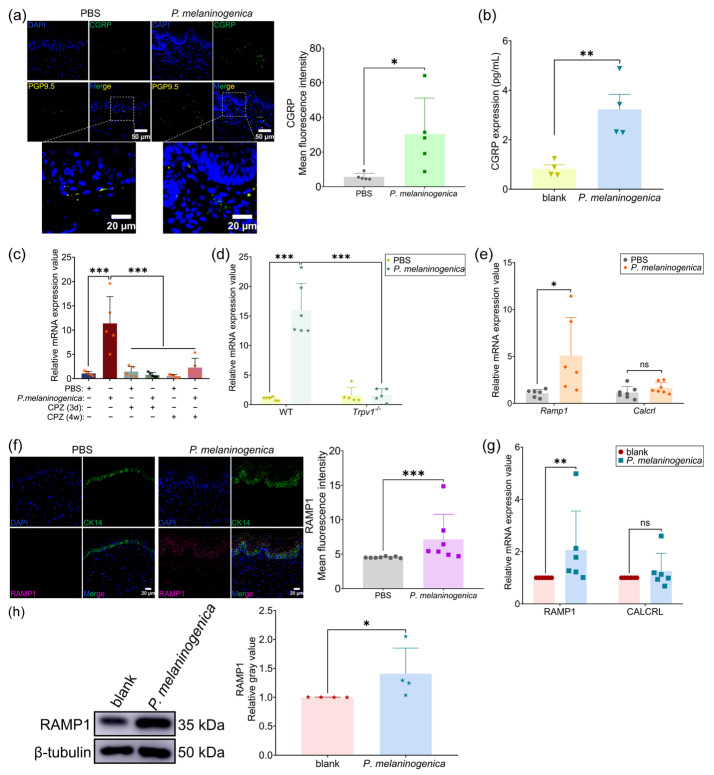
*P. melaninogenica* induced the release of CGRP via TRPV1. (**a**) Representative immunofluorescence images of buccal mucosal sections using CGRP and PGP9.5 antibodies from mice inoculated with PBS (*n* = 5) or *P. melaninogenica* (*n* = 5) and the quantitative analysis of CGRP and PGP9.5; (**b**) the protein level of CGRP in NHOKs following *P. melaninogenica* treatment by ELISA; (**c**) the mRNA expression of *Calca* in buccal mucosa of *P. melaninogenica*- and/or CPZ-treated mice. The number of symbols in the column represents the number of samples (*n* = 5/6); (**d**) The mRNA expression of *Calca* in buccal mucosa of WT and *Trpv1^−/−^* mice after *P. melaninogenica* inoculation. The number of symbols in the column represents the number of samples (*n* = 5/6); (**e**) the mRNA expression of *Ramp1* and *Calcrl* in buccal mucosa of mice inoculated by PBS (*n* = 6) or *P. melaninogenica* (*n* = 6/7); (**f**) representative immunofluorescence images of buccal mucosal sections using RAMP1 antibody from mice inoculated by PBS (*n* = 8) or *P. melaninogenica* (*n* = 7) and the quantitative analysis of RAMP1; (**g**) the mRNA expression of RAMP1 and CALCRL in NHOKs stimulated by *P. melaninogenica*; (**h**) western blotting and quantitative analysis of the protein level of RAMP1 in NHOKs stimulated by *P. melaninogenica*. * *p* < 0.05, ** *p* < 0.01, *** *p* < 0.001, ns: not significant.

Previous studies have found that, in neuronal cells, TRPV1 activation resulted in the release of its associated neuropeptide CGRP [29]. Firstly, we tested if *P. melaninogenica* can regulate CGRP release. It was shown that CGRP was distributed both in the epithelial layer and in the lamina propria after *P. melaninogenica* inoculation. The mean fluorescence intensity of CGRP was stronger. CGRP was co-localized with PGP9.5 or expressed alone in the epithelium or lamina propria (Figure 4a). In vitro, *P. melaninogenica* increased the protein level of CGRP in NHOKs (Figure 4b).

Then, we elucidated the role of TRPV1 in regulating CGRP. It was found that the mRNA level of *Calca* (calcitonin-related polypeptide alpha, encoding α-CGRP protein) was dramatically elevated after *P. melaninogenica* inoculation, and this elevation was significantly hampered by co-administration of CPZ or TRPV1 knockout (Figure 4c,d).

It was reported that heterodimers of calcitonin receptor-like receptor (CALCRL) and RAMP1 comprised the CGRP receptor and CGRP usually functioned in conjunction with them [37]. We examined their mRNA expressions and found that *Ramp1* was significantly increased in the buccal mucosa of mice after *P. melaninogenica* inoculation (Figure 4e). Also, the mean fluorescence intensity of RAMP1 and its co-location with CK14 were increased after *P. melaninogenica* inoculation (Figure 4f). To extend the in vitro findings above, it was also verified that elevated mRNA and protein levels of RAMP1 in NHOKs followed *P. melaninogenica* stimulation. (Figure 4g,h).

### 2.5. CGRP Was Highly Expressed in OLP and Enhanced IL-36γ Release by Binding to Its Receptor RAMP1 on Oral Keratinocytes

#### 2.5.1. CGRP and Its Receptor RAMP1 Were Highly Expressed in OLP

**Figure 5:**
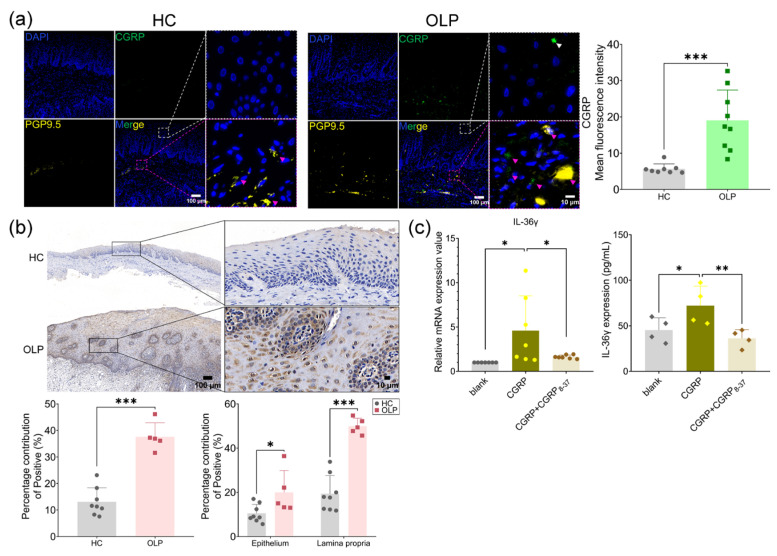
CGRP promoted IL-36γ production through binding to its receptor RAMP1 in NHOKs. (**a**) Representative immunofluorescence images of oral mucosal sections using CGRP and PGP9.5 antibodies in healthy controls (HCs) (*n* = 8) and OLP patients (*n* = 9) and the quantitative analysis of TRPV1. Purple arrows: CGRP clustering around PGP9.5; (**b**) immunohistochemistry and quantitative analysis of RAMP1 expression in healthy controls (HCs) (*n* = 8) and OLP patients (*n* = 5); (**c**) the mRNA and protein levels of IL-36γ in NHOKs treated with CGRP and CGRP_8-37_ through qPCR and ELISA. * *p* < 0.05, ** *p* < 0.01, *** *p* < 0.001.

Further, we detect the expression level of CGRP in OLP and the presence of co-localization with PGP9.5. As seen in Figure 5a, CGRP was expressed in both the epithelium and lamina propria in OLP but was only expressed in the lamina propria of normal tissue. CGRP was partially co-localized with PGP9.5, while CGRP clustering was seen around the expression of PGP9.5. Quantitative analysis showed that the mean fluorescence intensity of CGRP was significantly higher in OLP compared to healthy controls. Then, we detected the protein expression of RAMP1 in OLP and it was shown that RAMP1 was remarkably increased in OLP and its expression in the epithelial layer was also elevated in OLP (Figure 5b).

#### 2.5.2. CGRP Enhanced IL-36γ Release in NHOKs

Considering the presence of CGRP in the oral epithelium, next, we attempted to investigate the possible role of CGRP in the oral epithelium by treating NHOKs with CGRP and its receptor antagonist CGRP_8-37_. It was found that CGRP significantly increased the mRNA and protein levels of IL-36γ in NHOKs, which was impeded by the addition of CGRP_8-37_ (Figure 5c). These findings suggested that CGRP exerted its pro-inflammatory role through up-regulating IL-36γ.

### 2.6. CGRP Decreased P. melaninogenica Survival, Possibly Acting as an Antimicrobial Peptide

**Figure 6:**
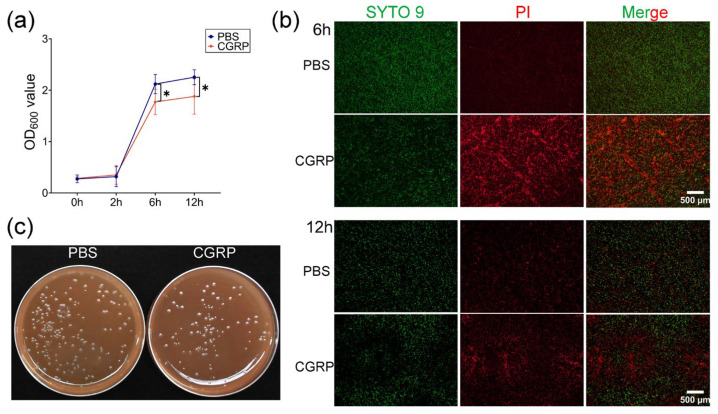
CGRP decreased the viability of *P. melaninogenica*. (**a**) The growth of *P. melaninogenica* treated by CGRP (5 × 10^−8^ M) was evaluated by measuring the OD_600_ value; (**b**) the damage of *P. melaninogenica* was tested by Live/Dead BacLight viability staining. The SYTO9 stain generally labels all bacteria in a population and propidium iodide (PI) labels damaged bacteria; (**c**) agar tests of *P. melaninogenica* culture after treatment with CGRP for 6 h. * *p* < 0.05.

A previous publication showed that CGRP exerted antimicrobial activity against *Escherichia coli* (*E. coli*), *Pseudomonas aeruginosa* (*P. aeruginosa*), *Candida albicans* (*C. albicans*), and *Streptococcus mutans* (*S. mutans*) [38]. To investigate whether CGRP could restrain *P. melaninogenica*, *P. melaninogenica* was treated with CGRP (5 × 10^−8^ M), and its growth was evaluated by measuring the OD_600_ value. The result displayed reduced bacterial growth at 6 h and 12 h after treatment with CGRP (Figure 6a). Live/Dead BacLight viability staining showed more dead *P. melaninogenica* after CGRP treatment (Figure 6b). Also, agar tests showed that the growth of *P. melaninogenica* was inhibited after treating it with CGRP for 6 h (Figure 6c). Accordingly, these findings revealed that CGRP displayed an antimicrobial role by decreasing the viability of *P. melaninogenica*.

### 2.7. IL-36γ Disrupted Oral Mucosal Epithelial Barrier via Down-Regulating ZO-1

**Figure 7:**
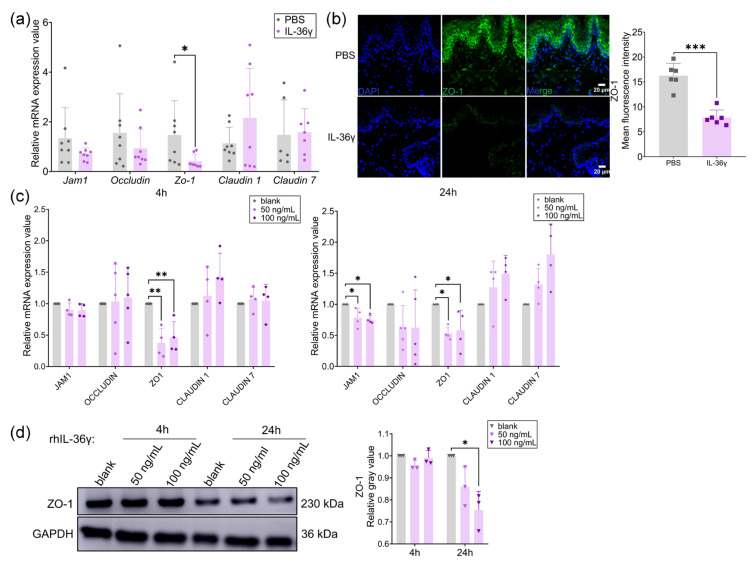
The oral mucosal epithelial barrier was disrupted by IL-36γ. (**a**) The mRNA expression of *Zo-1* in buccal mucosa of PBS- (*n* = 8) or rmIL-36γ-treated mice (*n* = 8); (**b**) representative immunofluorescence images of buccal mucosal sections using ZO-1 antibody from mice inoculated by PBS (*n* = 6) or rmIL-36γ (*n* = 6) and the quantitative analysis of ZO-1; (**c**) the mRNA expression of ZO-1 was detected in NHOKs treated with rhIL-36γ at the concentrations of 50 ng/mL and 100 ng/mL for 4 or 24 h; (**d**) the protein expression of ZO-1 in NHOKs treated with rhIL-36γ was measured by western blotting and its relative gray value was analyzed. * *p* < 0.05, ** *p* < 0.01, *** *p* < 0.001.

The oral epithelium serves as the first line of defense against bacterial invasion; hence, its barrier function is crucial [39]. With previous studies showing that IL-36γ can modulate the epithelial barrier [34], and considering the important role of the epithelial barrier in OLP [35,36], we explored the effect of IL-36γ on the oral epithelial barrier. rmIL-36γ was injected into the bilateral buccal mucosa of mice and the mRNA expression of some tight junction molecules was detected. Among these, *Zo1* showed reduced expression and weakened fluorescence intensity after rmIL-36γ stimulation compared to the control group (Figure 7a,b). Further, rhIL-36γ was used at different concentrations to stimulate NHOKs for 4 h or 24 h. It was shown that the mRNA level of ZO-1 was significantly decreased both in the short-term (4 h) and long-term (24 h) (Figure 7c), and its protein expression was decreased after 24 h of rhIL-36γ (Figure 7d). Overall, IL-36γ could disrupt the oral epithelial barrier by down-regulating ZO-1.

## 3. Discussion

In the current study, we have shown that TRPV1, CGRP, and its receptor RAMP1 were highly expressed in OLP. TRPV1 activation by capsaicin induced oral mucosal inflammation and regulated the *P. melaninogenica*-induced chronic inflammatory process. As a downstream neuropeptide of TRPV1, CGRP bound to its receptor to enhance the release of IL-36γ, which, in turn, destroyed the oral mucosal epithelial barrier by reducing ZO-1 expression. Moreover, CGRP decreased the viability of *P. melaninogenica*. Our findings offer new insights into the involvement of TRPV1-mediated oral inflammatory responses in the pathogenesis of OLP, suggesting that trigeminal neurons may play a crucial role in immunological and microbial homeostasis regulation.

The lack of animal models has been an obstacle in OLP research. In our preliminary studies, *P. melaninogenica* was found to be abundant in the buccal mucosa of OLP patients and further research revealed that it could invade the oral epithelium and even the lamina propria in OLP [14]. It also could activate the transcription of IL-1β, IL-6, and TNF-α in the NF-κB signaling pathway in macrophages [14]. We thus established a chronic inflammation model of the oral mucosa in mice using a local injection of *P. melaninogenica* for four weeks. According to our results, the expression levels of the pro-inflammatory cytokines (*Tnf-α*, *Il-1β*, and *Il-6*) in the *P. melaninogenica* group were found to be significantly elevated in the oral mucosa, which was consistent with the changes of TNF-α, IL-1β, and IL-6 in OLP. Histological examination found that inflammatory infiltration was obvious in the lamina propria of the oral mucosa of mice in the *P. melaninogenica* group, which suggested that *P. melaninogenica* was capable of inducing chronic oral mucosal inflammation in mice. However, the typical pathological characteristics of OLP, such as liquefied degeneration of epithelial basal cells and banded infiltration of subepithelial lymphocytes, were not detected. This may be explained by the fact that the pathogenesis of OLP is a multifactorial process [40] and that stimulation of *P. melaninogenica* alone may not be sufficient to establish a mouse model of OLP. Nonetheless, it can still mimic the chronic inflammatory state, to some extent, similar to the inflammatory manifestations of OLP (e.g., increased expression of some inflammatory cytokines, inflammatory infiltration, etc.). More exploration needs to be performed in the future.

Although TRPV1 was originally recognized as a sensory neuron-specific molecule, recent studies have revealed that this channel was also expressed in other cell types [41]. According to our results, the frequency of co-localization of TRPV1 with CK14 was increased in OLP and the expression of TRPV1 in the epithelium was significantly elevated in OLP, which is consistent with an early report [42]. This indicated that TRPV1 can be expressed in non-neuronal cells in the oral mucosa. Meanwhile, the oral epithelium could sense environmental changes, and both smoking and alcohol consumption could increase TRPV1 expression in the oral mucosa [43]. TRPV1 plays a key role in oral mucosal pain transduction [44]. Many scholars demonstrated an increased expression of TRPV1 in patients with burning mouth syndrome (BMS), which is a common oral mucosal disease characterized by a persistent burning sensation and pain lasting for more than three months without any local or systemic pathological changes [45,46]. The over-expression of TRPV1 was thought to be an important factor leading to the burning pain characteristic of BMS patients [46]. TRPV1 and TRPV1-expressing trigeminal nociceptors were thought to be the main cause of orthodontic force-induced pain behaviors and the main pathway for orthodontic pain transduction [44]. Accordingly, we hypothesized that TRPV1 may act as a sensor of pain and exogenous stimuli in the oral mucosa and that the elevated expression in OLP makes TRPV1 more sensitive to changes in the external environment, which partially explains the clinical manifestation of pain after eating irritating food in OLP patients.

TRPV1 activation exerted pro- or anti-inflammatory effects in an environment-dependent and tissue-specific manner. A recent discovery indicated that the activation of TRPV1+ neurons alone was sufficient to initiate the immune system despite the absence of exogenous factors and its “preventive” activation has been found to positively affect the body through initiating innate type 17 preventive immunity [47]. TRPV1^+^ sensory nerves accelerated corneal wound healing by suppressing inflammation [48]. Conversely, blockade of TRPV1 evidently prevented mitochondrial damage and the inflammatory response in cigarette smoke-induced airway epithelial cell injury [49]. Herein, in the normal mucosa, activating TRPV1 by exogenous stimuli promoted inflammatory infiltration and the expression of inflammatory factors, suggesting that aberrant activation of TRPV1 may be a pro-inflammatory factor as regards the oral mucosa, which triggers inflammation and affects homeostasis maintenance in the oral mucosa.

From this study, TRPV1 expression was significantly elevated in trigeminal ganglia during *P. melaninogenica* invasion of the oral mucosa, suggesting that *P. melaninogenica* invasion may stimulate local nerve endings, which leads to the transduction of stimulation signals to the trigeminal ganglia. In sensory neurons, TRPV1 may contribute to triggering and regulating innate defense against Gram-negative bacterial infection [50]. This also indicated that TRPV1 activation in trigeminal ganglia may trigger a series of responses in response to *P. melaninogenica* invasion, such as inducing an inflammatory response and promoting the release of neuropeptides from neurons [29].

The nervous system, immune system, and microbial pathogens cooperate intimately, and CGRP plays a pivotal role in their cooperation. Sensory neurons activated by *Streptococcus pyogenes* released CGRP into infected sites, and CGRP has been found to prevent neutrophils from effectively eliminating bacteria [51]. Similarly, trigeminal ganglia neurons released more CGRP when exposed to *P. aeruginosa* and, in turn, CGRP mediated the transformation of macrophages [52]. Toxin B from *Clostridioides difficile* leads to the release of the neuropeptides SP and CGRP from neurons and pro-inflammatory cytokines from pericytes, resulting in neurogenic inflammation [53]. Another report showed that CGRP release following *Porphyromonas gingivalis* (*P. gingivalis*) LPS stimulation in trigeminal sensory neurons was inhibited by the TRPV1 antagonist CPZ [53]. Our findings are similar to, but different from, previous studies. The similarities were that we confirmed that *P. melaninogenica* was able to cause increased release of CGRP and that antagonizing TRPV1 inhibited CGRP expression. Differently, while previous studies suggested that bacteria stimulate CGRP release by activating neurons, in our study, *P. melaninogenica* was found to activate TRPV1 on both neurons and oral keratinocytes in the oral mucosa, and the immunofluorescence result also showed that CGRP co-localized not only with PGP9.5 but also appeared in the epithelium alone after *P. melaninogenica* stimulation. Regarding this phenomenon, there are two possible reasons: one, CGRP was secreted into the epithelium after release by neurons, and two, CGRP was simultaneously produced by non-neuronal cells other than neurons. Our results revealed that *P. melaninogenica* promoted CGRP release in oral keratinocytes. Keratinocytes, like neurons, are derived from ectoderm and exhibit a variety of neurochemical properties [54,55]. Voltage-gated sodium channels were expressed on keratinocytes and can be involved in ATP release to produce nociception [56]. In human skin keratinocytes, *Propionibacterium acnes* activated TRPV1 via TLR4, which, in turn, induced CGRP release [57]. Accordingly, it was demonstrated that neurons were not the only cells releasing CGRP after *P. melaninogenica* invasion of oral mucosa and that keratinocytes were also involved in this process. This showed that keratinocytes were not only regulated neurogenically by CGRP released from sensory endings, but also possibly by its own secretion. On the whole, the interplay between *P. melaninogenica* and CGRP was a complicated process in the microenvironment of oral mucosal inflammation. Upon invasion of the oral mucosa, *P. melaninogenica* was able to trigger CGRP release, further promoting local inflammatory processes. Secreted CGRP, in turn, performed its function as an antimicrobial peptide to inhibit the growth of *P. melaninogenica* and became one of the body’s defense armies against *P. melaninogenica* invasion.

**Figure 8:**
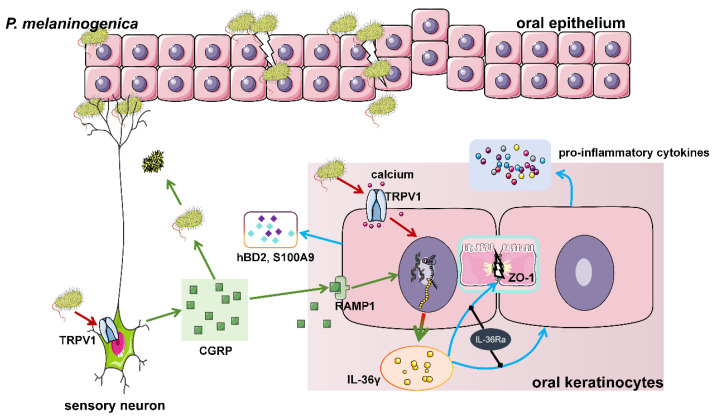
A schematic model of TRPV1/CGRP signaling in oral inflammatory responses induced by *P. melaninogenica* in oral lichen planus. *P. melaninogenica* invasion into the oral mucosa activated the TRPV1 channel, promoted calcium influx, and increased IL-36γ expression in oral keratinocytes. IL-36γ caused lower expression of ZO-1 and disruption of the epithelial barrier, resulting in a greater level of *P. melaninogenica* invasion. On the other hand, *P. melaninogenica* promoted the release of CGRP and the expression of RAMP1 in oral keratinocytes, which positively regulated the release of IL-36γ. Meanwhile, CGRP also destroyed *P. melaninogenica* survival by acting as an antimicrobial peptide. The schematic model was designed using images available from Servier Medical Art (http://smart.servier.com/ (accessed on 2 March 2021)).

In this study, we observed that *P. melaninogenica* induced a pro-inflammatory response in oral keratinocytes by enhancing the expression of OLP-associated inflammatory cytokines and that blocking TRPV1 suppressed *P. melaninogenica*-induced IL-36γ production, which suggested that IL-36γ was a downstream cytokine closely related to TRPV1 activation and that TRPV1 can regulate *P. melaninogenica*-induced oral mucosal inflammation via IL-36γ. The role of IL-36γ in OLP has been gradually revealed by researchers in the last few years. IL-36γ was recently found to exhibit increased expression in OLP and it exacerbated inflammatory progression in oral keratinocytes by promoting the production of pro-inflammatory cytokines [15,35]. Vo PT et al. have suggested that IL-36γ can serve as a biomarker to distinguish the diagnosis of OLP from other oral mucosal diseases [35]. Meanwhile, the IL-36 family has been implicated in the regulation of epithelial barrier function. Exogenous rhIL-36α was shown to disrupt the corneal epithelial barrier by reducing the expression of ZO-1 and occludin in human corneal epithelial cells, while IL-36RA and IL-38 were found to protect against such disruption [58]. The IL-36R pathway significantly increased intestinal permeability and down-regulated the expression of the tight junction protein occludin in cases of gut injury [33]. In our study, it was seen that IL-36γ leads to barrier dysfunction by down-regulating the expression of ZO-1 in NHOKs. It is presumed that IL-36γ can exacerbate the pathogenesis of OLP by disrupting the oral epithelial barrier and promoting more invasion of harmful microbes (including *P. melaninogenica*). In summary, *P. melaninogenica* may be involved in the pathogenesis of OLP by mediating crosstalk between oral keratinocytes and neurons via the TRPV1/CGRP-IL-36γ pathway (Figure 8).

Our study still has some limitations. Previous studies have highlighted some structural and biophysical similarities between neuropeptides and host defense peptides, which allowed neuropeptides to cause microbial cell lysis and death [59]. Karim et al. reported that CGRP could directly defend against certain pathogens such as *E. coli*, *P. aeruginosa*, *C. albicans,* and *S. mutans* at different minimal inhibitory concentrations (MICs) ranging from 2.1 μg/mL to more than 500 μg/mL [38]. In our study, CGRP influenced the survival of *P. melaninogenica* and led to increased bacterial death at a concentration of 5 × 10^−8^ M. Another study showed that CGRP induced a strong stimulation of *Staphylococcus epidermidis* virulence with a low threshold (<10^−12^ M) [60]. It was suggested that the efficient concentration of CGRP was significantly different depending on the bacterial strains. However, the precise mechanisms underlying this phenomenon remain unclear and warrant further investigation. In this study, only *P. melaninogenica* (ATCC^®^ 25845™) was employed and no control strains (e.g., other *P. melaninogenica* strains or other oral microbial species, such as *P. gingivalis* and herpes viruses) were established, and, although this did not interfere the current conclusions, we will improve it in future studies to obtain more findings. Additionally, the differences in pain intensity in different types of OLP were not taken into account, while the limited sample size hampered a more in-depth exploration.

## 4. Materials and Methods

### 4.1. Sample Collections

The nine subjects diagnosed with OLP by experienced oral mucosal professionals and confirmed by histopathologic examination were enrolled at the Department of Oral Medicine, School of Stomatology, Tongji University, and their related information was presented in Supplementary Table S1. Inclusion criteria were referred to in Cheng’s previous study, and include both clinical and pathological criteria [61,62]. The inclusion criteria were as follows: two clinicians with expertise in oral mucosal diseases confirmed the clinical examination, as well as histopathological diagnosis in accordance with the World Health Organization’s modified version of the OLP diagnostic criteria. The exclusive criteria were as follows: (a) with tumor or systematic diseases; (b) with removable or fixed denture in the mouth; (c) salivary glands diseases; (d) periodontal depth more than 4 mm, with aggressive caries, untreated pulp disease or periapical problems; (e) smoking history; (f) no drug abuse (especially no antibiotics or any drug which might lead to lichenoid reaction in the last 1 month, no glucocorticoids or immunomodifier in the last 6 months); (g) females in menstruation, pregnancy or lactation; (h) concurrently suffering from other oral mucosal diseases. The study protocol was approved by the Ethics Committee of the Affiliated Stomatology Hospital of Tongji University (No. 2018-002). Informed consent was obtained from all subjects before this study.

### 4.2. Cell Culture

Normal human oral keratinocytes (NHOKs) were taken and cultured according to our previous protocol but from different subjects [15]. The study protocol was reviewed and approved by the Ethics Committee of the Affiliated Stomatology Hospital of Tongji University (Ethics Approval No. [2021]-SR-01). NHOKs were taken from the superfluous buccal mucosa during minor surgical procedures. After overnight digestion with dispase II (Roche, Atlanta, GA, USA), the lower adipose tissue was removed, and then the epithelial tissue was cut into pieces. These pieces were digested using 0.25% trypsin (Gibco, Grand Island, NY, USA) for 4 min, then centrifuged at 1000 rpm for 5 min, and finally resuspended using OKM medium (Sciencell, Carlsbad, CA, USA). Cells were maintained at 37 °C in a humidified atmosphere of 5% CO_2_.

### 4.3. Cell Treatments

NHOKs were treated with recombinant human IL-36γ (rhIL-36γ) (CM77) (novoprotein, Shanghai, China) at concentrations of 50 and 100 ng/mL. TRPV1 agonist capsaicin (M9962, Abmole, Houston, TX, USA) was used to treat NHOKs at a concentration of 10^−12^ M. TRPV1 antagonist capsazepine (CPZ) (211280, Sigma-Aldrich, Burlington, MA, USA) was used to treat NHOKs at a concentration of 10^−9^ M. CGRP (C0167, Sigma-Aldrich, USA) and CGRP_8-37_ (SCP0060, Sigma-Aldrich, USA) were used to treat NHOKs at a concentration of 10^−12^ M and 10^−9^ M, separately.

### 4.4. Bacteria Culture

*P. melaninogenica* (ATCC^®^ 25845™) was cultured with Anaerobic Basal Broth (Oxoid CM0957 Oxoid, Ogdensburg, NY, USA). *P. melaninogenica* cells from overnight cultures were diluted in fresh broth and harvested during early exponential growth. After centrifugation, the *P. melaninogenica* pellet was suspended in an antibiotic-free medium and was adjusted to OD_600_ = 1.0. The cells were inoculated with bacterial suspensions at 37 °C. NHOKs were stimulated by *P. melaninogenica* at MOI 5 for 4 h.

### 4.5. Fluo-4 AM Staining

For Ca^2+^ imaging, after *P. melaninogenica* treatment, cells were labeled with Fluo-4 AM (Beyotime, Shanghai, China) for 45 min at 37 °C, washed, and incubated for 20 min. Then, the images were scanned by an Olympus fluorescence microscope (APX100, Tokyo, Japan).

### 4.6. Immunofluorescence and Immunohistochemistry

Tissue samples from OLP patients or healthy controls and buccal mucosa of mice were fixed with 10% buffered formalin and embedded in paraffin. The paraffin sections were baked at 65 °C for 1 h, then were submerged in xylene for 15 min at room temperature for deparaffinization. The sections were rehydrated by submerging them in 100%, 95%, 80%, 70%, and 50% ethanol solution at room temperature. Then, sections were submerged in antigen repair buffer for antigen retrieval. The sections were incubated for 30 min with a blocking solution. Then, a primary antibody was added and the sections were incubated overnight at 4 °C. Secondary antibody staining was carried out for 1 h at room temperature. For the sections used for immunofluorescence, the images were scanned (Pannoramic MIDI, 3DHISTECH Ltd., Budapest, Hungary). For the sections used for immunohistochemistry, the sections were then treated with DAB staining and hematoxylin counterstaining. Finally, dehydration and sealing with neutral gum were performed. The images were captured by an Olympus fluorescence microscope (APX100). All images were quantified by Image J (v1.8.0). The information about antibodies is listed in Table 1.

### 4.7. RNA Extraction and RT-qPCR

Total RNA was extracted using TRIzol Reagents (Life Technologies, Carlsbad, CA, USA) according to the manufacturer’s instructions and was reversely transcribed into cDNA by the PrimeScript™ 1st Strand cDNA Synthesis Kit (Takara, Shiga, Japan). A two-step qPCR was performed using a PrimeScript™ RT reagent Kit (Takara, Japan) and TB Green Premix Ex Taq (Takara, Japan) according to the manufacturer’s instructions. The GAPDH was used as internal control and the relative expression levels were determined using the comparative CT method (2^−∆∆CT^). Primers were designed from Primer Premier 6.0. All information about the primers is listed in Supplementary Table S2.

### 4.8. Enzyme-Linked Immunosorbent Assay (ELISA)

We collected and concentrated the cell supernatant after treatment. ELISA was performed with commercial ELISA kits according to the manufacturer’s instructions (Human IL-36γ ELISA Kit (PI635, Beyotime, Shanghai, China), Human CGRP ELISA Kit (CSB-E08210h, CUSABIO, Houston, TX, USA). Preparation step: equilibrate the kits at room temperature for at least 20 min before using and preparing and then prepare the standards. Formal experiments: Standards or samples were added to the 96 wells and incubated for 2 h at RT. The biotinylated antibody was added and incubated for 1 h at RT. Then, horseradish peroxidase-labeled Streptavidin was added and incubated for 20 min at RT in a dark room. Afterward, the TMB solution was added and incubated for 20 min at RT in a dark room; finally, the termination solution was added and the absorbance value at 450 nm was measured immediately using a microplate reader (SpectraMax M5, Molecular Devices, San Jose, CA, USA).

### 4.9. Western Blotting

Cells and tissues were collected and lysed in ice-cold RIPA lysis buffer (Beyotime, Shanghai, China). The supernatant was collected after centrifugation at 12,000 rpm for 15 min. Total protein was separated using from 6% to 15% SDS-PAGE gels, and the proteins in the gel were transferred to PVDF membranes (Millipore, St. Louis, MO, USA). The membranes were incubated with primary antibodies overnight at 4 °C, followed by incubation of the membrane with appropriate horseradish peroxidase-conjugated secondary antibodies at room temperature for 1 h. The bands were visualized with enhanced chemiluminescence reagent BeyoECL Plus (Beyotime, Shanghai, China). The images were quantified by Image J. Detailed information about the primary antibodies is listed in Table 2.

### 4.10. Mice

A total of 130 C57BL/6J mice (6 weeks old, body weight: 22 g) were used as the study objects. *Trpv1*-knockout (*Trpv1^−/−^*) mice and littermate wild-type (WT) mice were purchased from Shanghai Model Organisms Center, Inc. (Shanghai, China), using CRISPR/Cas9 technology. The littermates were grouped depending on the purpose of the experiments. As the human oral cavity itself is a germy environment, we did not make germ-free mice to perform bacteriological-related experiments. We performed the injection under sterile conditions. Before performing each injection, the buccal mucosa of the mice was sterilized. All mice were sacrificed by intraperitoneal injection of an overdose of 2% pentobarbital sodium. The study protocol was approved by the Ethics Committee of the Affiliated Stomatology Hospital of Tongji University ([2021]-DW-02).

#### 4.10.1. *P. melaninogenica* Infection Model

The mice were anesthetized by intraperitoneal injection of 1% pentobarbital sodium (8 mL/kg body weight). *P. melaninogenica* was adjusted to OD_600_ = 0.6 and the mice were injected with 50 μL of bacterial suspension or sterile PBS into the buccal mucosa, respectively, every other day. After four weeks, the mice were sacrificed, and the buccal tissue and trigeminal ganglion were taken for further analysis. Part of the buccal samples were immediately stored at −80 °C for RNA extraction and part of the buccal samples and trigeminal ganglion were placed in 4% paraformaldehyde for immunofluorescence.

#### 4.10.2. Capsaicin Injection

Capsaicin was injected into the buccal mucosa (10^−6^ M, 100 μL/per mouse). The control group received equivalent dose injections with sterile PBS. After four weeks, the mice were sacrificed, and the buccal tissue was taken for RNA analysis.

#### 4.10.3. Pharmacological Blocking TRPV1 Channel

**Figure 9:**
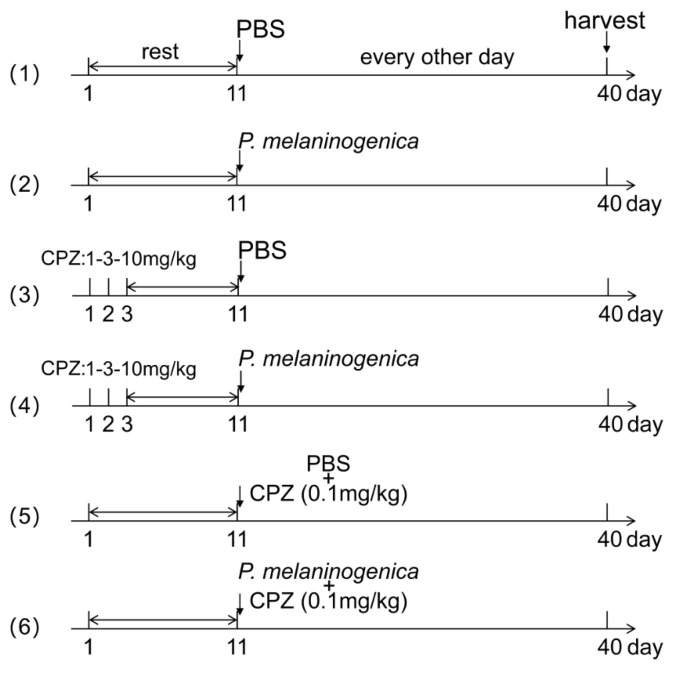
*P. melaninogenica* was injected into the buccal mucosa of mice combined with the TRPV1 antagonist capsazepine (CPZ). Group 1 and 2: Starting on day 11, 50 μL of sterile PBS or *P. melaninogenica* suspension was injected into the buccal mucosa, respectively, every other day. Group 3 and 4: CPZ was injected into the buccal mucosa of mice for three consecutive days at concentrations of 1, 3, and 10 mg/kg, respectively, and the mice were left to rest for a week. On day 11, 50 μL of sterile PBS or *P. melaninogenica* suspension was injected into the buccal mucosa respectively every other day. Group 5 and 6: Starting on day 11, CPZ (2.5 mg/kg, i.d.) was injected into the buccal mucosa of mice 30 min beforehand and then 50 μL of sterile PBS or *P. melaninogenica* suspension was injected into the buccal mucosa, respectively, every other day.

We used two separate methods to block TRPV1 expression according to the references [63,64]. (a) CPZ) was injected into the buccal mucosa of mice for three consecutive days at concentrations of 1, 3, and 10 mg/kg, respectively, and rest for a week before inoculating with *P. melaninogenica* or PBS. We designated this method as CPZ 3d. (b) The mice were pretreated with CPZ (2.5 mg/kg, i.d.) for 30 min, which was designated as CPZ 4w. More details are shown in Figure 9. Mice were sacrificed and the buccal tissue was taken for RNA analysis and hematoxylin and eosin staining (HE).

#### 4.10.4. IL-36γ Injection

Recombinant murine IL-36γ (rmIL-36γ) (BioLegend 552806, San Diego, CA, USA) was carefully injected into the buccal mucosa (200 ng/mL, 40 ng/per mouse) every other day. The control group received injections with sterile PBS. After four weeks, the mice were sacrificed, and the buccal tissue was taken for RNA and immunofluorescence analysis.

### 4.11. HE

After the mice were sacrificed, the buccal mucosa was immediately removed and postfixed by overnight immersion in a 4% PFA solution. Sections were dehydrated in ethanol, embedded in paraffin, and stained with hematoxylin and eosin. Sections were visualized and photographed using an Olympus microscope (APX100).

### 4.12. Detection of P. melaninogenica Viability

Bacterial growth viability was measured using a spectrophotometer and agar tests. Also, the viability of CGRP-treated bacteria was assessed using the LIVE/DEAD™ BacLight™ kit (L13152, ThermoFisher Scientific, Waltham, MA, USA) according to the manufacturer’s protocol. Images were acquired by an Olympus fluorescence microscope (APX100).

### 4.13. Statistical Analysis

Statistical analysis was performed using Prism software (version 8.4.2, GraphPad Software, San Diego, CA, USA). Data were presented as the mean ± standard deviation. For statistical analysis between two groups, firstly, normality and lognormality tests were carried out. A non-parametric two-tailed Mann–Whitney test was used for data that did not have a normal distribution and an unpaired Student’s *t*-test for normally distributed data. For statistical analysis among three or more groups, the homogeneity of variance test was performed by an F-test. Comparisons among them were made based on an analysis of variance and a corresponding post hoc test to compare the averages between each group. *p* < 0.05 was considered to indicate a statistically significant difference. In all graphs, * *p* < 0.05, ** *p* < 0.01, and *** *p* < 0.001.

## Figures and Tables

**Table 1:** Detailed information about primary antibodies in immunofluorescence.

Antibodies	Concentration	Source	Identifier
Cytokeratin 14 (CK14)	1:200	Proteintech	60320-1-Ig
TRPV1	1:200	Abcepta	AP13988a
CGRP	1:400	CST	14959T
PGP9.5	1:500	Proteintech	14730-1-AP
RAMP1	1:200	Proteintech	10327-1-AP
ZO-1	1:1000	Proteintech	21773-1-AP

**Table 2:** Detailed information about primary antibodies in western blotting.

Antibodies	Concentration	Source	Identifier
TRPV1	1:1000	Abcepta	AP13988a
RAMP1	1:1000	Proteintech	10327-1-AP
ZO-1	1:2000	Proteintech	66452-1-Ig
GAPDH	1:8000	Proteintech	10494-1-AP
β-tubulin	1:8000	Proteintech	10094-1-AP

## Data Availability

The datasets generated during and/or analyzed during the current study are available from the corresponding author upon reasonable request.

## Supplementary Materials

The supporting information can be downloaded at https://www.mdpi.com/article/10.3390/ijms26020662/s1.
