# Supplementary material for: The Role of TRPV1/CGRP Pathway Activated by Prevotella melaninogenica in Pathogenesis of Oral Lichen Planus
Source: Int J Mol Sci. 2025 Jan 14;26(2):662. doi: 10.3390/ijms26020662 (PMC11766222; doi:10.3390/ijms26020662)
Supplement: Supplementary file 1 [file ijms-26-00662-s001.zip › ijms-3341146-supplementary.pdf]

Table S1. Sample information from patients with oral lichen planus (OLP).

No.	age	sex	Type	Sites
OLP1	42	M	Atrophic	Tongue
OLP2	56	W	Reticular	Buccal mucosa
OLP 3	66	W	Atrophic	Buccal mucosa
OLP 4	53	W	Erosive	Buccal mucosa, Gingiva, Tongue
OLP 5	54	M	Reticular	Buccal mucosa, Tongue
OLP 6	60	W	Erosive	Buccal mucosa
OLP 7	55	M	Atrophic	Gingiva
OLP 8	38	M	Reticular	Buccal mucosa
OLP 9	58	W	Erosive	Buccal mucosa

Table S2. Primer sequences in the manuscript.

	Forward primer (5'-3')	Reverse primer (5'-3')
TNF-α	AGAACTCACTGGGGCCTACA	AGGAAGGCCTAAGGTCCACT
IL-1β	CTGTCCTGCGTGTTGAAAGA	TTCTGCTTGAGAGGTGCTGA
IL-6	CCACCGGGAACGAAAGAGAA	GAGAAGGCAACTGGACCGAA
IL-36γ	GGTGCTGAGACAACCACACT	CTGAAGGGTCCACACTTGCT
CALCA	ATCAGAGACACTGCCCAGC	TTGGAAGCCCATGACACCTC
RAMP1	GAGGCACGCTTGCTTCAC	AGGGCACCGTAGTTAGCCTC
CALCRL	CCCACCTTGCTTGTGGGTAA	TCAAGACCCAGTCCAGCTCT
JAM1	CCTAGTGCCCGAAGTGAAGG	CGGTAGCACCTGAGTAAGGC
OCCLUDIN	GGTCTAGGACGCAGCAGATT	GCCTGGATGACATGGCTGAT
ZO-1	GTGCTGGCTTGGTCTGTTTG	TGACGCTGGGTGATAGGGAT
CLAUDIN 1	CTGGGAGGTGCCCTACTTTG	ACACGTAGTCTTTCCCGCTG
CLAUDIN 7	CCCTCCACCTTTTGTTTGCC	GCACAGGGAGTAGGATACGC
GAPDH	GGACCTGACCTGCCGTCTAG	GTAGCCCAGGATGCCCTTGA
Trpv1	CCGTCAGCTCTGTTGTCACT	GATCATAGAGCCTTGGGGGC
Tnf-α	CCCATATACCTGGGAGGAGTCTTC	CATTCCCTTCACAGAGCAATGAC
Il-1β	GAAATGCCACCTTTTGACAGTG	TGGATGCTCTCATCAGGACAG
Il-6	CTGCAAGAGACTTCCATCCAG	AGTGGTATAGACAGGTCTGTTGG
Il-36γ	AAGTCGGTGGGATTGGAGTG	TCAGGCCAATGGCTGGAAAT
Calca	CGCTCACCAGGAAGGCATCA	CCCACACCGCTTAGATCTGG

Ramp1	TCGCAGGGTCTCTAACCAGA	GACCATGCAGTAGAGGCCAA
Calcrl	TCCGGGAAACTCTTGTGCAG	GACCTGAGTGTTAGACCATCCA
Jam1	TGTGGGATTGGGCAAGAGTC	TAGAGTAGCTGGCACCCCAT
Occludin	CCGGCCGCCAAGGTTC	CTTTCAAAAGGCCTCACGGA
Zo-1	TGACCTCTGCAGCAATAAAGC	ACAGAAATCGTGCTGATGTGC
Claudin 1	CCACCATTGGCATGAAGTGC	CTGGCATTGATGGGGGTCAA
Claudin 7	CAAGGGCCCGCATACTTTCT	TGGTTCCAGACAAAAGCGGT
Gapdh	TGATGGGTGTGAACCACGAG	AGTGATGGCATGGACTGTGG
